# Melatonin Ameliorates Hepatic Ferroptosis in NAFLD by Inhibiting ER Stress via the MT2/cAMP/PKA/IRE1 Signaling Pathway

**DOI:** 10.7150/ijbs.85883

**Published:** 2023-07-31

**Authors:** Qingyun Guan, Zixu Wang, Keyu Hu, Jing Cao, Yulan Dong, Yaoxing Chen

**Affiliations:** 1 College of Veterinary Medicine, China Agricultural University, Haidian, Beijing 100193, China.; 2 Department of Nutrition and Health, China Agricultural University, Haidian, Beijing 100193, China.

**Keywords:** Melatonin, NAFLD, Liver, Ferroptosis, MT2, ER stress, IRE1

## Abstract

Ferroptosis, an iron-dependent cell death form, has recently been observed in the development of non-alcoholic fatty liver disease (NAFLD). Melatonin (Mel) shows potential benefits for preventing and treating liver diseases. Whether and how Mel ameliorates hepatic ferroptosis in NAFLD is not fully understood. Here we established a mouse model of NAFLD induced by long-term high-fat diet (HFD) feeding. We found that Mel treatment ameliorated global metabolic abnormalities and inhibited the progression of NAFLD in mice. Most importantly, Mel supplementation significantly improved HFD-induced iron homeostasis disorders in the liver, including iron overload and ferritin transport disorders. For another, Mel ameliorated HFD-induced hepatic lipid peroxidation. The recuperative role of exogenous Mel on hepatocyte ferroptosis was also observed in PA- or Erastin-treated HepG2 cells. Mechanistically, MT2, but not MT1, was involved in the effect of Mel. Furthermore, Mel treatment inhibited HFD or Erastin-activated ER stress and activated the PKA/IRE1 signaling pathway. Co-expression of p-PKA and p-IRE1 was enhanced by the MT2 antagonist. Inhibitions of PKA and IRE1 respectively improved hepatocyte ferroptosis, and activations of cAMP/PKA reversed Mel's effect on ferroptosis. Collectively, these findings suggest that exogenous Mel inhibits hepatic ferroptosis in NAFLD by ameliorating ER stress through the MT2/cAMP/PKA/IRE1 pathway, proving that Mel is a promising candidate drug for the treatment of hepatic ferroptosis in NAFLD.

## Introduction

Non-alcoholic fatty liver disease (NAFLD) is emerging as a growing public health challenge. NAFLD's epidemiological and demographic traits mimic those of obesity prevalence 1, and its prevalence is rising alarmingly fast, with the latest prevalence rate of 34% in Asia 2 and an incidence of 6% to 35% in the adult population globally 3. NAFLD begins with steatosis and can proceed to nonalcoholic steatohepatitis (NASH) and, eventually, fibrosis and cirrhosis. There are currently no effective drug treatments for NAFLD. Future research must address information gaps to lessen the risk burden given the burden of NAFLD 4. An important development is that alternative types of cell death, other than apoptosis, necrosis, or charring, may be involved in NAFLD-related hepatotoxicity.

A hazardous buildup of lipid peroxide triggers the recently identified form of cell death known as ferroptosis, which governs cell death in an iron-dependent manner 5. Iron metabolism disorders and lipid peroxidation are the main characteristics of ferroptosis 6. Mechanistically, the regulation of ferroptosis involves different pathways, such as the p53 pathway, the glutamine pathway, and the KEAP1-NRF2 pathway 7. In recent years, the role of hepatic ferroptosis in the onset and development of NAFLD has been emphasized. Hepatocyte ferroptosis may worsen NAFLD by raising the likelihood of hepatocyte swelling, inflammation, and fibrosis, thus speeding up the progress from simple steatosis to NASH 8, 9. Therefore, inhibiting hepatic ferroptosis is likely to be a therapeutic target for NAFLD.

Ferroptosis is linked to endoplasmic reticulum (ER) stress. Activation of ER signaling has been reported to mediate ferroptosis 10, 11. Meanwhile, abnormal accumulation of iron would also induce unfolded protein response (UPR) and subsequently activate ER stress pathway. Among the three typical molecular branches of UPR, inositol-requiring enzyme 1 (IRE1) is the most conserved ER stress sensor. IRE1 dimerizes and autophosphorylates in response to ER stress, activating its kinase and endoribonuclease activities 12. Protein kinase R-like endoplasmic reticulum kinase (PERK) is another classical ER stress pathway. The significance of ER stress-mediated by PERK in ferroptosis is well-established 13, while the mediated role of IRE1 remains uncertain. Overall, more investigation is required into the existing connection between hepatic ferroptosis and ER stress in NAFLD.

Melatonin (Mel), a potent antioxidant, has sparked widespread interest due to its numerous physiological effects. In mammals, type 1A (MT1) and type 1B (MT2) membrane receptors are the main mechanisms by which Mel operates 14. Recent research supports the view that Mel is an excellent ferroptosis inhibitor, inhibiting ferroptosis in the hippocampus 15 and lung 16. Mel's impact on hepatic ferroptosis in NAFLD has not been extensively characterized, nevertheless. Studies on the relationship between Mel and NAFLD are limited, but the available data suggest a beneficial role in inhibiting NAFLD progression. Given that Mel contributes to suppressing oxidative stress in the liver, we speculate that Mel is related to hepatic ferroptosis in NAFLD. Therefore, in this study, we explored the inhibitory effect and mechanism of Mel on hepatic ferroptosis to prove whether Mel is a promising candidate drug for the treatment of hepatic ferroptosis in NAFLD.

## Materials and Methods

### Animal treatment

The "Guidelines for the Care and Use of Laboratory Animals" published by the Animal Welfare Committee of the Agricultural Research Organization, China Agricultural University, served as the foundation for the experiment (Approval No.AW92303202-2-2). Three-week-old male C57BL/6 J mice (Charles River Co. Ltd., Beijing, China) were raised under ideal conditions (at 21±1°C, 50% relative humidity, and a 12 h: 12 h light/dark cycle) and were given access to food and water without restriction. After one week of accommodation, the mice were typically split into two groups, which fed either a normal chow diet (NCD, Charles River Co. Ltd., Beijing, China) or a high-fat diet (HFD, 60% of energy from lipids; Beijing HFK Bioscience Co., Ltd., Beijing, China). Mice from the HFD group were then split into two groups (HFD and HFD+Mel) on average after 10 weeks. Mice in the HFD+Mel group received intraperitoneal injections of the Mel therapy (40 mg/kg body weight, dissolved in anhydrous ethanol) for 6 weeks, while the mice in the other two groups received the same anhydrous ethanol injection dosage. The dosage of Mel was selected based on previous studies from ours 15, 17 or others 18-20, with slight modifications.

### Glucose and insulin tolerance test

For a glucose tolerance test, mice were fasted for 12 h before receiving an intraperitoneal injection of glucose (1 g/kg body weight, Sigma, St. Louis, MO, USA). A portable glucose monitor (Aike, Lingrui, Hangzhou, China) was used to collect blood samples from the tip of the tail at 0, 15, 30, 60, 90, and 120 minutes following glucose injection. GraphPad Prism (version 9.4, GraphPad Software Inc., San Diego, CA, USA) was used to compute and statistically analyze the area under the curve. Similarly, insulin (0.5 IU/kg, Novolin R, Novo Nordisk, Denmark) was administered intraperitoneally following a 6 h overnight fast, and blood glucose was monitored as previously mentioned.

### Blood biochemical analysis

Commercially available kits were used to measure the levels of plasma triglyceride (TG), total cholesterol (TC), fasting blood glucose, alanine aminotransferase (ALT), and aspartate aminotransferase (AST) (Jiancheng Bioengineering Institute, Nanjing, China). The directions are rigorously followed, and each procedure is carried out three times.

### Histological staining

Harvested livers were dehydrated using a succession of progressively graded alcohols for 48 h before being embedded in paraffin. Sections of the liver (5 μm) were cut using semiautomated rotator microtome and were stained with hematoxylin and Eosindye (H&E). Meanwhile, Masson's trichrome staining and Sirius Red staining were performed using modified stain kits (Solarbio Life Science, Beijing, China) to assess fibrosis. Frozen liver sections (10 μm) were stained with Oil Red O (Solarbio Life Science). To measure the iron in the liver, sections of the liver were stained with a Prussian blue iron stain kit (with nuclear fast red, Solarbio Life Science) or an improved Perls' Prussian blue staining method by Zhang et al 21. All samples were examined and captured under a microscope (BX51, Olympus, Tokyo, Japan). Experimenters were blinded to groups when assessing the staining results statistically. The percentage of locations that were iron-positive was used to represent the outcome. Experienced physiologists used the steatosis, ballooning, and inflammation scores to assess the NAFLD activity score 22.

### Measurements of lipid peroxidation and antioxidant activity

Liver tissues were immediately homogenized and clarified lysates were produced by centrifugation at 4 °C (12,000 g for 10 min). Protein concentrations were determined using a protein assay kit (CW0014, CWBIO, Beijing, China). Five commercial kits were used in accordance with the manufacturer's instructions to measure the amount of malondialdehyde (MDA, S0131S; Beyotime, Beijing, China), the total antioxidant capability (T-AOC, S0116; Beyotime), the activities of glutathione peroxidase (GSH-Px, S0056; Beyotime), superoxide dismutase (SOD, S0101S; Beyotime), and catalase (CAT, S0051; Beyotime) in the liver.

### Immunohistochemistry and immunofluorescence

Primary antibodies were treated overnight at 4 °C with paraffin-embedded liver slices or fixed cells for the immunohistochemistry (IHC) staining or the immunofluorescence assay. See the Supplementary Material for more detailed information.

### Quantitative real-time (RT)-PCR analysis

TRIzol reagent (R401-01-AA; Vazyme, Nanjing, China) was used to extract total mRNA from liver tissues. See the Supplementary Material for more detailed information on processes of reverse transcription and RT-PCR. The primers were listed in Table S1 of the Supplementary Material.

### Western blot assay

RIPA lysate (CW2333S, CWBIO) with 1% protease inhibitor (CW2200S, CWBIO) and 1% phosphatase inhibitor (CW2383S, CWBIO) was used to lyse proteins from liver tissues or HepG2 cells. The lysate was centrifuged at 12,000 g for 15 min at 4 °C. Using a protein assay kit (CW0014, CWBIO), the supernatant's protein concentration was measured. See the Supplementary Material for more detailed information.

### Cell culture and treatment

HepG2 cells were cultured in high-glucose DMEM containing 10% fetal bovine serum (FBS) and 1% penicillin/streptomycin in a CO_2_ incubator (5% CO_2_, 37℃). Cells were grown in DMEM with 10% FBS (complete media) for 6 h after being plated in 6 or 96 well plates, respectively, prior to drug treatment. After that, the cells were grown for 12 h in DMEM without FBS (basal medium). See the Supplementary Material for more detailed information.

### Statistical analysis

GraphPad Prism (version 9.4, GraphPad Software Inc., San Diego, CA, USA) was used to analyze the data, which are shown as the mean ± standard error of the mean (SEM). One-way ANOVA was used to statistically evaluate group differences. The cutoff for statistical significance was *p* < 0.05.

## Results

### Mel treatment improved global metabolic abnormalities in HFD-fed mice

**Figure 1:**
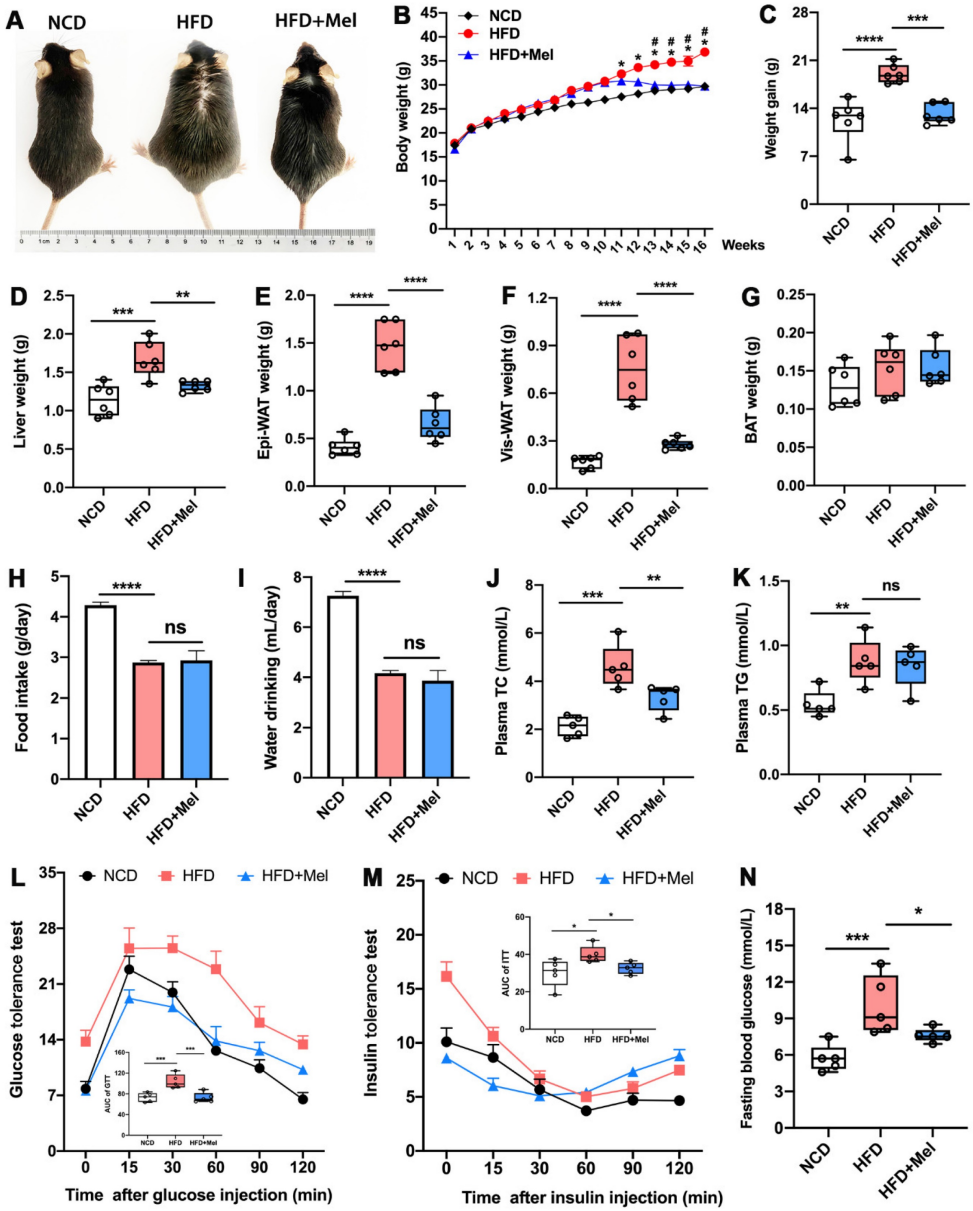
** Mel improved global metabolic abnormalities in HFD-fed mice.** (A) Mice images. (B) Body weight (n = 6). (C) Weight gain (n = 6). (D) Liver weight (n = 6). (E) Epi-WAT weight (n = 6). (F) Vis-WAT weight (n = 6). (G) BAT weight (n = 6). (H) Food intake. (I) Water drinking. (J) Plasma TC (n = 5). (K) Plasma TG (n = 5). (L) Glucose tolerance test (n = 5). (M) Insulin tolerance test (n = 5). (N) Fasting blood glucose (n = 5). The results are presented as the mean ± SEM. Differences were assessed by one-way ANOVA. *p < 0.05, **p < 0.01, ***p < 0.001, and ****p < 0.0001. *p < 0.05 in (B) compared to the NCD group. ^#^*p* < 0.05 in (B) compared to the HFD group.

We started by looking into Mel's impact on the overall metabolic anomalies in mice given long-term HFD feeding. Mel supplementation significantly prevented body weight gain and obesity induced by HFD (Figure 1A-C). Except for brown adipose tissue (BAT), the weights of the liver, epididymal white adipose tissue (Epi-WAT), and visceral white adipose tissue (Vis-WAT) elevated by HFD also decreased after Mel therapy (Figure 1D-G). As previously reported, HFD markedly inhibited the daily food intake and water intake (Figure 1H, I) of mice compared with NCD, but Mel therapy did not change the diet. Mel consistently improved HFD-induced dyslipidemia, resulting in lower plasma TC concentration (Figure 1J). Mel also decreased fasting blood glucose levels and repaired glucose tolerance and insulin sensitivity that had been damaged by HFD (Figure 1L-N). In conclusion, Mel improved the overall metabolic abnormalities of the HFD mice.

### Mel treatment inhibited the progression of NAFLD in mice

**Figure 2:**
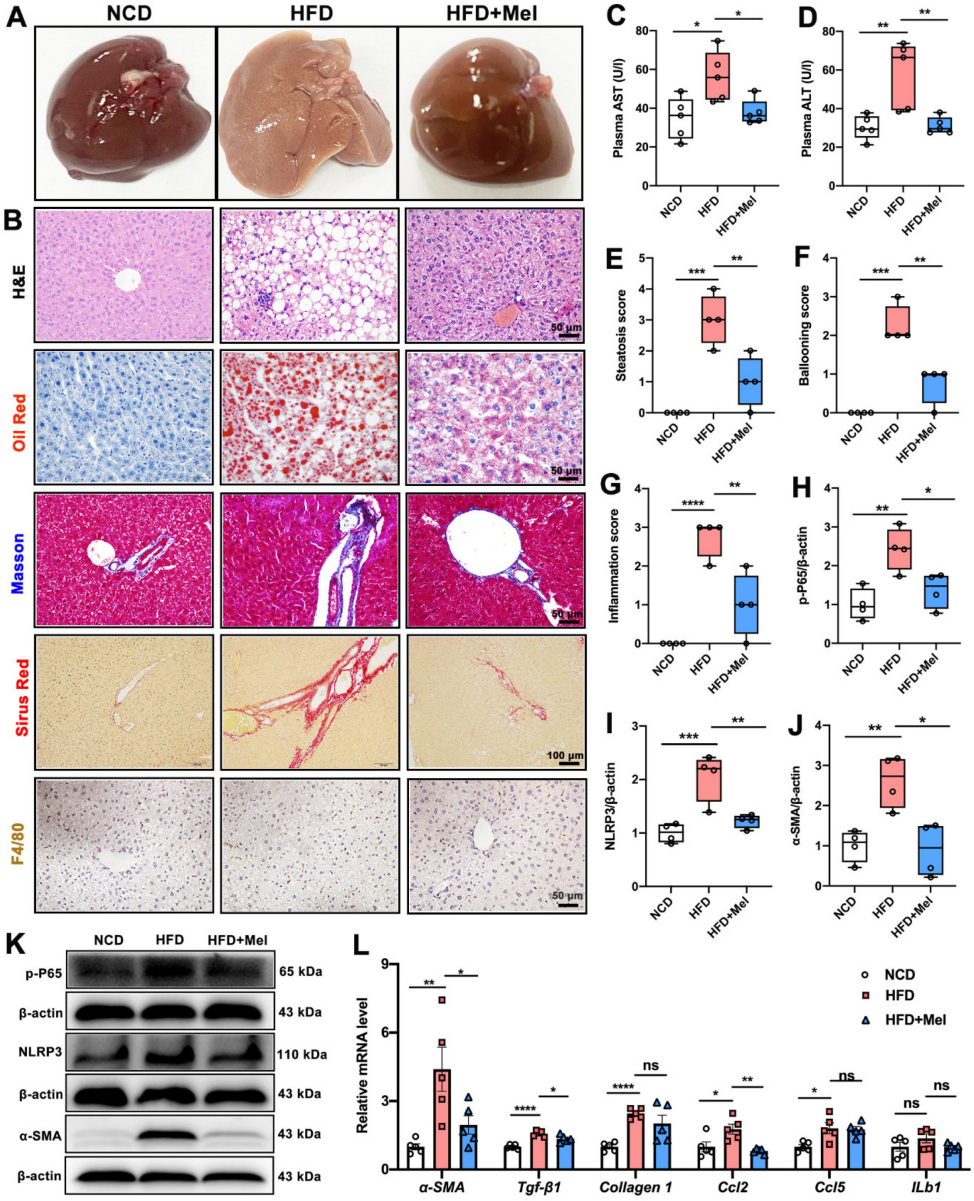
** Mel inhibited the progression of NAFLD in mice induced by long-term HFD feeding.** (A) Liver image. (B) Histology analysis including H&E (scale: 50 μm), Oil Red (scale: 50 μm), Masson (scale: 50 μm), Sirius Red (scale: 100 μm), and F4/80 IHC staining (scale: 50 μm). (C) Plasma AST (n = 5). (D) Plasma ALT (n = 5). (E) Steatosis score (n = 4). (F) Ballooning score (n = 4). (G) Inflammation score (n = 4). (H-K) Relative protein levels of p-P65, NLRP3, and α-SMA (n = 4). (L) Relative mRNA levels of *α-SMA, Tgf-β1, Collagen1, Ccl2, Ccl5*, and *ILb1* (n = 5). The results are presented as the mean ± SEM. Differences were assessed by one-way ANOVA. *p < 0.05, **p < 0.01, ***p < 0.001, and ****p < 0.0001.

The effect of Mel on the progression of NAFLD was next explored. HFD significantly induced liver enlargement, soft texture, light yellow color, vague section structure, and a greasy feeling, whereas Mel treatment restored them to the control state (Figure 2A). Multiple morphological analyses consistently showed that Mel significantly reduced HFD-induced hepatic steatosis, fibrosis, and F4/80 labeled inflammatory infiltration (Figure 2B), which was accompanied by a drop in plasma AST and ALT (Figure 2C, D), liver steatosis score, ballooning score, and inflammation score (Figures 2E-G). Furthermore, Mel treatment reduced protein levels of p-P65, NLRP3, and α-SMA involved in inflammation or fibrogenesis (Figure 2H-K). Additionally, RT-PCR results revealed that Mel decreased mRNA levels of *α-SMA, Tgf-β1*, and *Ccl2* raised by HFD (Figure 2L). As a result, it was concluded that Mel treatment slowed the progression of NFALD in mice fed with HFD.

### Mel alleviated hepatic iron homeostasis dysregulation in NAFLD

**Figure 3:**
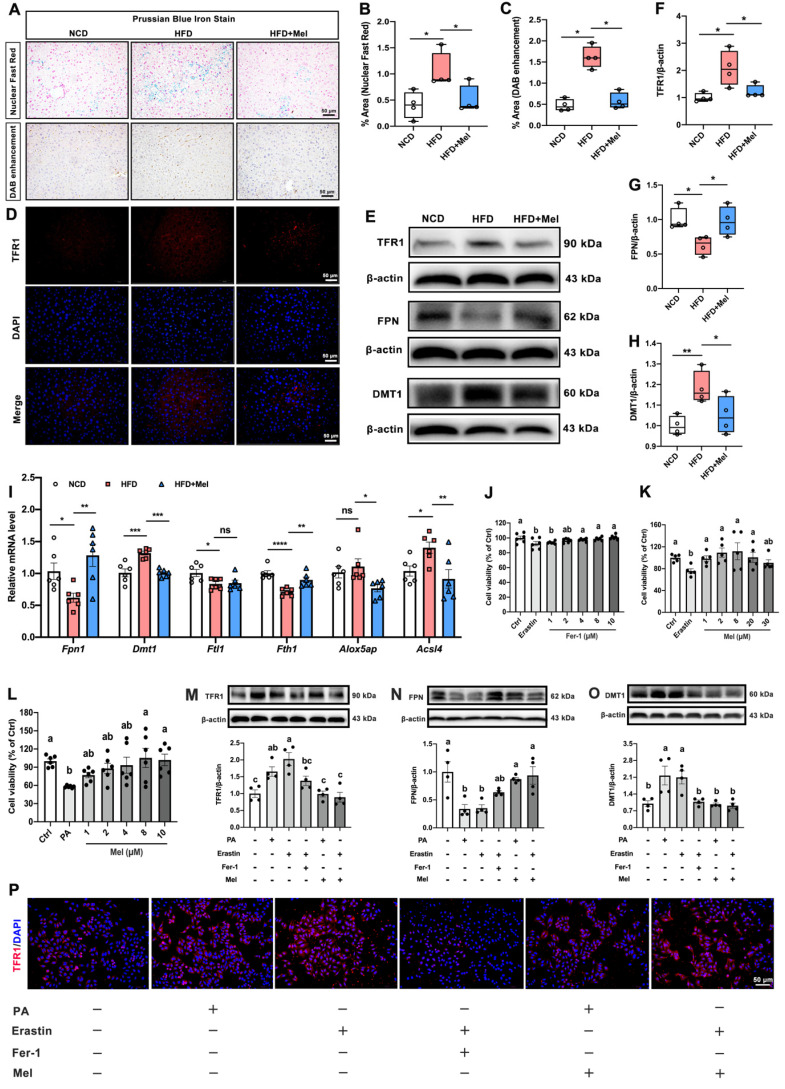
** Mel alleviated hepatic iron homeostasis dysregulation in NAFLD.** (A) Prussian blue iron staining (scale: 50 μm). (B) % area of Prussian blue staining with Nuclear Fast Red (n = 4). (C) % area of Prussian blue staining with DAB enhancement (n = 4). (D) Immunofluorescence analysis of TFR1 in the liver (scale: 50 μm). (E-H) Relative protein levels of TFR1, FPN, and DMT1 in the liver (n = 4). (I) Relative mRNA levels of *Fpn1, Dmt1, Ftl1, Fth1, Alox5ap,* and *Acsl4* in the liver (n = 6). (J-L) Cell viability (% of Ctrl) (n = 5-6). (M-O) Relative protein levels of TFR1, FPN, and DMT1 in HepG2 cells (n = 4). (P) Immunofluorescence analysis of TFR1 in HepG2 cells (scale: 50 μm). The results are presented as the mean ± SEM. Differences were assessed by one-way ANOVA. Values without the same superscript letter were significantly different (*p* < 0.05); those with the same letter do not differ significantly (*p* ≥ 0.05). *p < 0.05, **p < 0.01, ***p < 0.001, and ****p < 0.0001.

To test whether Mel could improve hepatic ferroptosis in NAFLD, we first assessed iron homeostasis in the liver. Prussian blue iron staining (including the nuclear fast red method and DAB enhancement method) consistently showed that the intracellular iron ions in the liver (blue or brown positive areas) were increased by HFD; however, Mel intervention prevented the buildup of hepatic iron ions (Figure 3A-C). Following that, the transport of iron ions was evaluated. Mel reversed HFD-induced increases in protein levels of transferrin receptor 1 (TFR1, Figure 3D-F) and divalent metal transporter 1 (DMT1, Figure 3E, H) and decreased in ferroportin (FPN, Figure 3E, G) protein in the liver. In addition, Mel restored hepatic mRNA levels of *Fpn1*, *Dmt1*, ferritin heavy chain 1 (Fth1), and acyl-CoA synthetase long-chain family member 4 (*Acsl4*) (Figure 3I).

We grew HepG2 cells and exposed them to Erastin (1 μM) or palmitic acid (PA, 250 μM) with or without Mel (1 μM) or Ferrostatin-1 (Fer-1, 2 μM) to further investigate the function of Mel in preventing hepatocytes from ferroptosis. Fer-1 and Mel could protect Erastin- or PA-induced cell death in hepatocytes (Figure 3J-L). Intriguingly, PA could mimic the effect of Erastin, inducing an increase of TFR1 and DMT1 and a decrease of FPN proteins significantly in HepG2 cells. In contrast, Mel pretreatment could mirror the effect of Fer-1, restoring iron transporters disorders brought on by PA and Erastin (Figure 3M-P). These results suggested that Mel could alleviate hepatic iron homeostasis and thus improved iron overload.

### Mel alleviated hepatic lipid peroxidation and mitochondrial dysfunction in NAFLD

**Figure 4:**
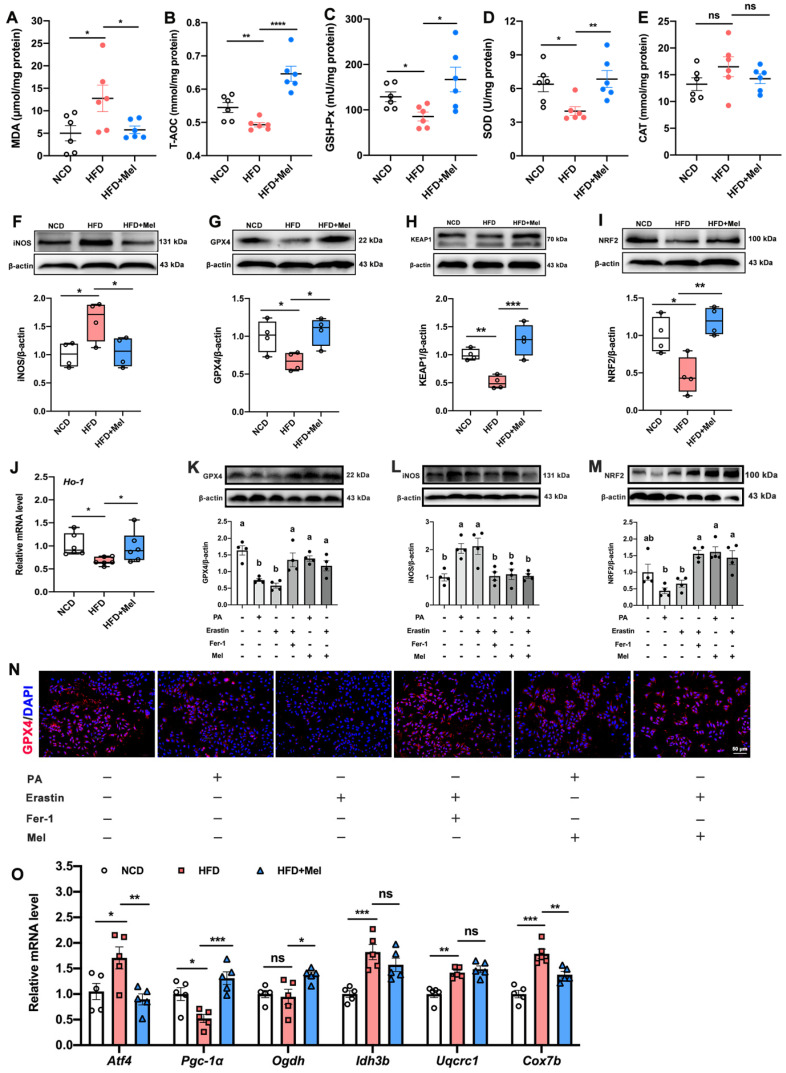
** Mel alleviated hepatic lipid peroxidation and mitochondrial dysfunction in NAFLD.** (A-E) The levels of MDA, T-AOC, GSH-Px, SOD, and CAT in the liver (*n* = 6). (F-I) Relative protein levels of iNOS, GPX4, KEAP1, and NRF2 in the liver (n = 4). (J) Relative mRNA levels of Ho-1 in the liver (n = 6). (K-M) Relative protein levels of GPX4, iNOS, and NRF2 in HepG2 cells (n = 4). (N) Immunofluorescence analysis of GPX4 in HepG2 cells (scale: 50 μm). (O) Relative mRNA levels of *Atf4, Pgc-1α, Odgh, Idh3b, Uqcrc1,* and *Cox7b* in the liver (n = 5). The results are presented as the mean ± SEM. Differences were assessed by one-way ANOVA. Values without the same superscript letter were significantly different (*p* < 0.05); those with the same letter do not differ significantly (*p* ≥ 0.05). *p < 0.05, **p < 0.01, ***p < 0.001, and ****p < 0.0001.

Another hallmark of ferroptosis is lipid peroxidation. Compared with NCD, HFD had a considerably higher concentration of MDA (Figure 4A) and a lower concentration of T-AOC (Figure 4B), GSH-Px (Figure 4C), and SOD (Figure 4D) in the liver. However, these changes were reversed by Mel supplementation. Besides, Mel dramatically reduced the HFD-induced increase of iNOS, and decline in GPX4, KEAP1, NRF2, and Ho-1 in the liver (Figure 4F-J). *In vitro* studies consistently confirmed Mel treatment could improve PA- or Erastin-induced lipid peroxidation in hepatocytes (Figure 4K-N). Since the lipid peroxidation process is associated with mitochondrial dysfunction, we also examined a series of genes implicated in mitochondrial function. Mitochondrial oxidative gene (activating transcription factor 4, ATF4), mitochondrial oxidative phosphorylation gene (Cox7b, cytochrome c oxidase subunit VIIb), and mitochondrial biosynthesis gene (Pgc-1α, peroxisome proliferator-activated receptor-γ coactivator-1α) could be significantly restored by the treatment of Mel (Figure 4O).

### Mel improved hepatic ferroptosis through the MT2 but not the MT1 signaling pathway

**Figure 5:**
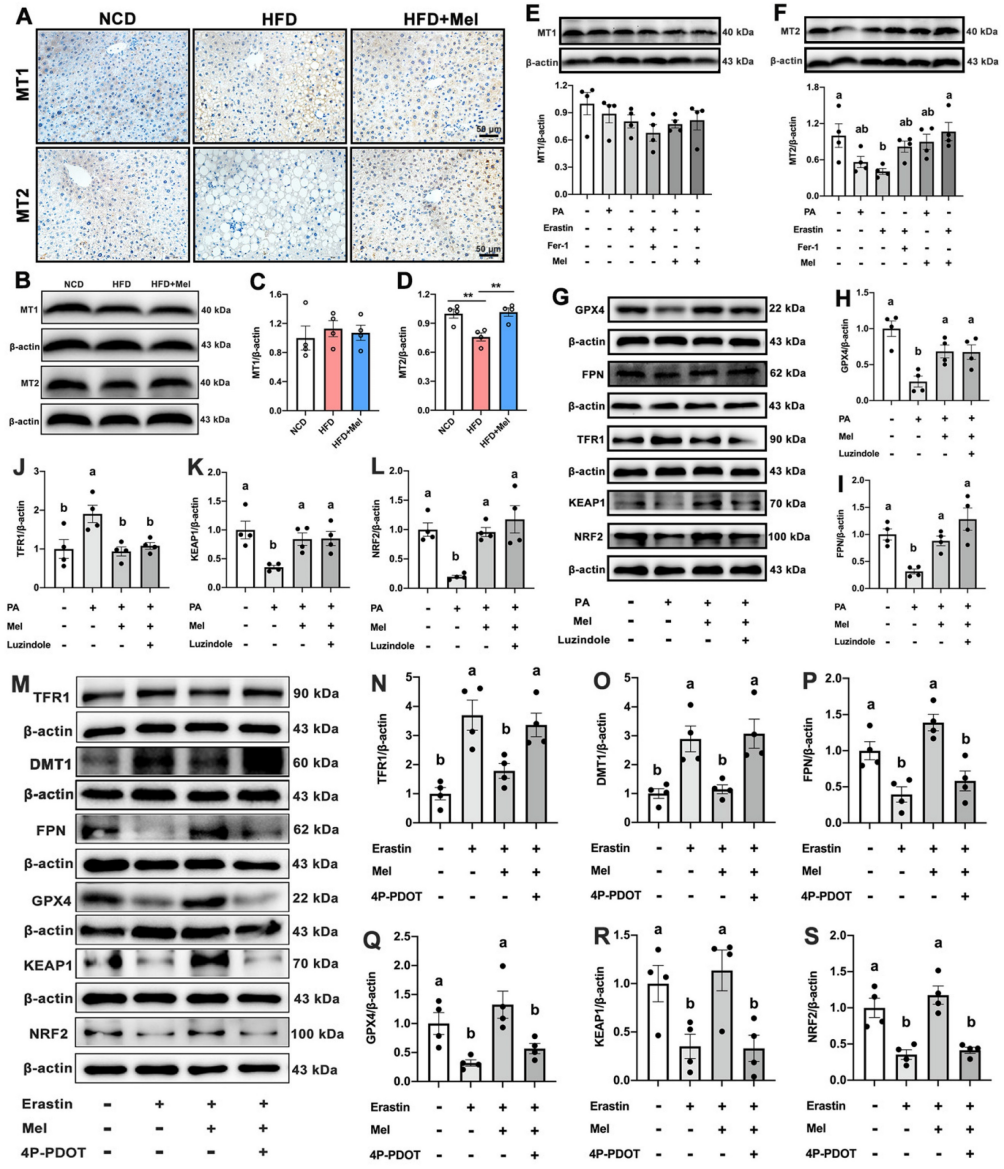
** Mel improved hepatic ferroptosis through MT2.** (A) IHC analysis of MT1 and MT2 in the liver (scale: 50 μm). (B-D) Relative protein levels of MT1 and MT2 in the liver (n = 4). (E-F) Relative protein levels of MT1 and MT2 in HepG2 cells (n = 4). (G-L) Relative protein levels of GPX4, FPN, TFR1, KEAP1, and NRF2 in HepG2 cells treated with PA, Mel, or Luzindole (n = 4). (M-S) Relative protein levels of TFR1, DMT1, FPN, GPX4, KEAP1, and NRF2 in HepG2 cells treated with Erastin, Mel, or 4P-PDOT (n = 4). The results are presented as the mean ± SEM. Differences were assessed by one-way ANOVA. Values without the same superscript letter were significantly different (*p* < 0.05); those with the same letter do not differ significantly (*p* ≥ 0.05). **p < 0.01.

Membrane receptors MT1 or MT2 are recognized to be the main mechanisms via which Mel acts. As shown in Figure 5A, MT1 and MT2 were widely expressed on the liver cell membrane. There was no statistically significant difference in MT1 expression (Figure 5B, C). Mel was however able to restore the HFD-decreased MT2 protein level (Figure 5B, D). In line with the *in vivo* findings, PA, Erastin, or Mel therapy had no effect on the protein level of MT1 (Figure 5E). In contrast, Mel pretreatment counteracted the reduction in MT2 induced by Erastin (Figure 5F). Therefore, Mel's defense against hepatic ferroptosis may depend more on MT2 than MT1. To rule out the action of MT1, we treated HepG2 cells with an MT1 inhibitor (Luzindole). As respected, Luzindole was unable to block Mel's effect on PA-induced changes in GPX4, FPN, TFR1, KEAP1, and NRF2 (Figure 5G-L). Nonetheless, 4P-PDOT pretreatment abolished Mel's relieving impact on Erastin-induced ferroptosis (Figure 5M-S). Therefore, Mel may improve hepatic ferroptosis through MT2.

### Mel ameliorated hepatic ferroptosis by inhibiting ER stress through the MT2/c-AMP/PKA/IRE1 signaling pathway

**Figure 6:**
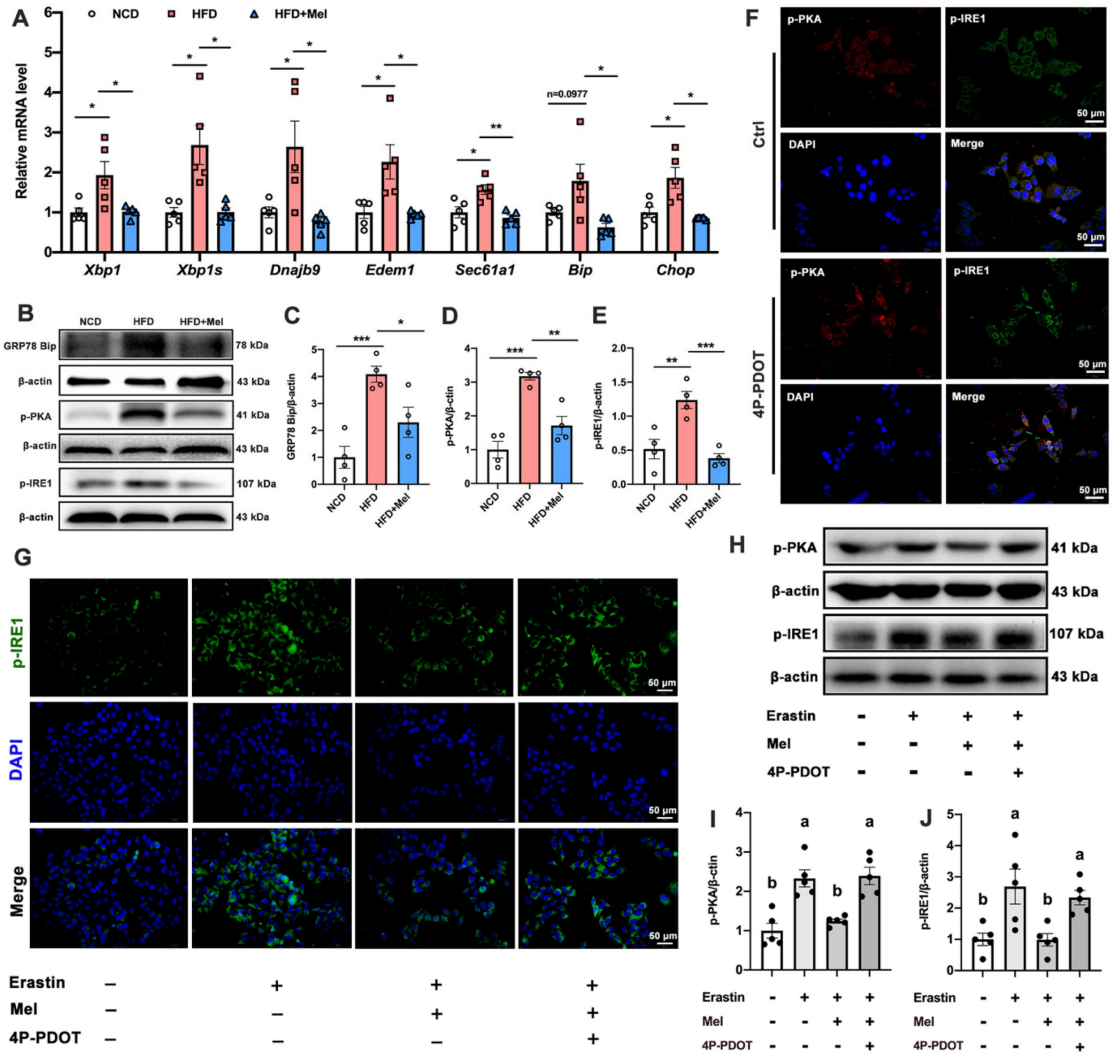
**Mel/MT2 inhibited hepatic ER stress induced by HFD or Erastin.** (A) Relative mRNA levels of *Xbp1, Xbp1s, Dnajb9, Edem1, Sec61a1, Bip,* and *Chop* in the liver (n = 5). (B-E) Relative protein levels of GRP78 Bip, p-PKA, and p-IRE1 in the liver (n = 4). (F) Co-expression of p-PKA (red), p-IER1 (green), and DAPI (blue) in HepG2 cells treated with or without 4p-PDOT (scale: 50 μm). (G) Immunofluorescence analysis of p-IER1 in HepG2 cells (scale: 50 μm). (H-J) Relative protein levels of p-PKA and p-IRE1 in HepG2 cells (n = 5). The results are presented as the mean ± SEM. Differences were assessed by one-way ANOVA. Values without the same superscript letter were significantly different (*p* < 0.05); those with the same letter do not differ significantly (*p* ≥ 0.05). *p < 0.05, **p < 0.01, and ***p < 0.001.

Hepatocyte ER stress is potentially involved in ferroptosis in the liver. We next examined the effect of ER stress on hepatic ferroptosis and the role of Mel in it. The relative mRNA expression levels of X-box binding protein 1 (XBP1), spliced XBP1 (XBP1s), and representative XBP1-dependent UPR target genes (dnaJ heat shock protein family member B9 [Dnajb9], er-degradation-enhancing α-mannosidase-like protein 1 [Edem1], SEC61 transposon α1 subunit [Sec61a1], bound immunoglobulin [Bip], and CCAAT/enhancer-binding protein homologous protein [Chop]) were significantly upregulated in the liver induced by HFD, while Mel could restore them to the NCD levels (Figure 6A). Similarly, Mel could reverse the expression of GRP78 Bip, p-PKA, and p-IRE1 proteins increased by HFD (Figure 6B-E). These results suggested that the ER stress pathway was activated in NAFLD, and Mel could ease it.

**Figure 7:**
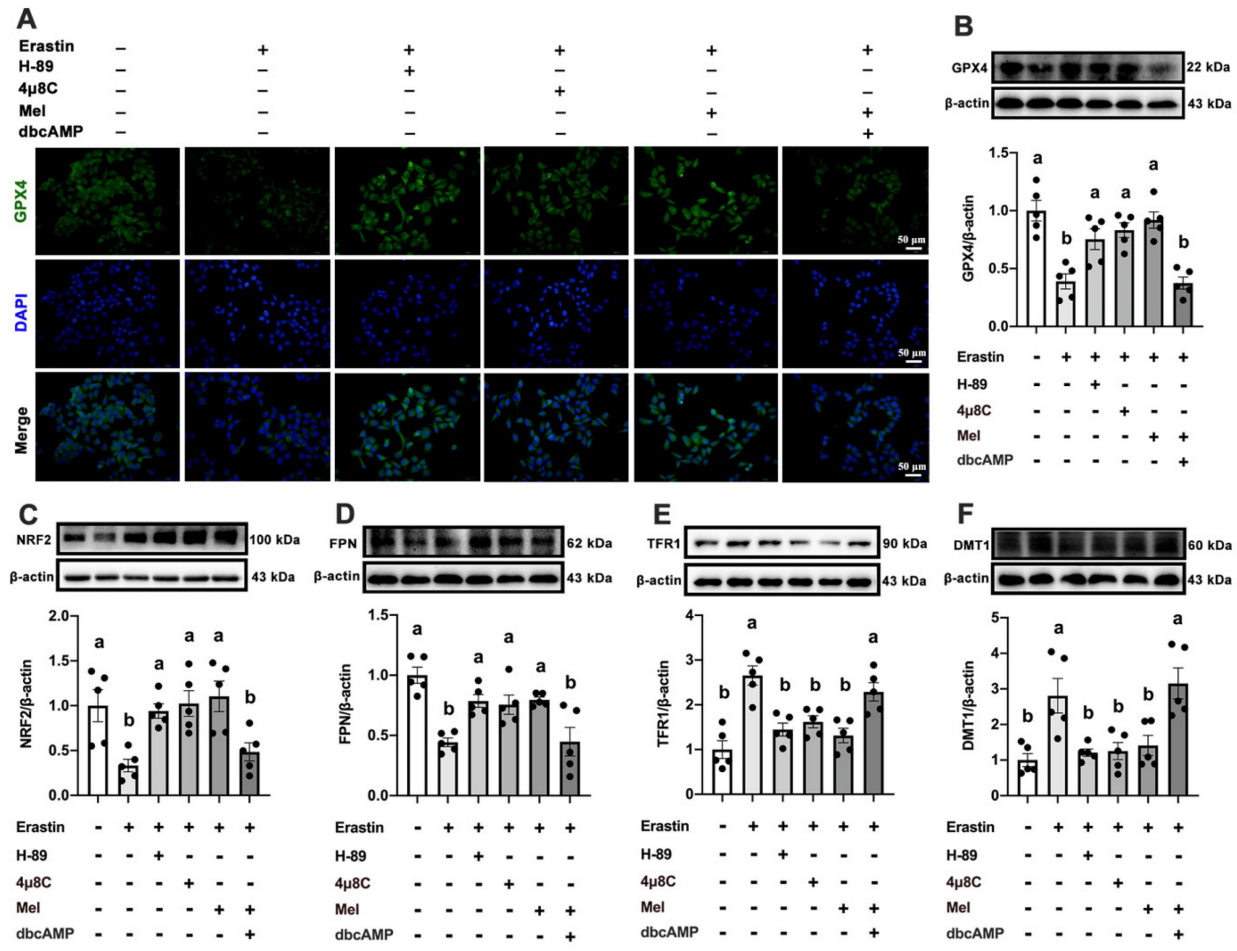
** Mel ameliorated hepatic ferroptosis by inhibiting ER stress through the MT2/cAMP/PKA/IRE1 signaling pathway.** (A) Immunofluorescence analysis of GPX4 in HepG2 cells treated with Erastin, H-89, 4μ8C, Mel, or dbcAMP (scale: 50 μm). (B-F) Relative protein levels of GPX4, NRF2, FPN, TFR1, and DMT1 in HepG2 cells (n = 5). The results are presented as the mean ± SEM. Differences were assessed by one-way ANOVA. Values without the same superscript letter were significantly different (*p* < 0.05); those with the same letter do not differ significantly (*p* ≥ 0.05).

MT2 is a G protein-coupled receptor. Protein kinase A (PKA) is a cAMP-dependent protein kinase phosphorylated IRE1 in Ser724 23. Next, we investigated the effect of Mel/MT2 in activating the cAMP/PKA/IRE1 signaling pathway. p-PKA was colocalized with p-IRE1 in the hepatocyte, which was enhanced by 4P-PDOT (Figure 6F). p-PKA and p-IRE1 levels in hepatocytes were considerably increased after treatment with Erastin. Mel addition could significantly restore them, while pretreatment with 4P-PDOT negated Mel's effects (Figure 6G-J). Hence, the loss of MT2 in the liver significantly activates the PKA/IRE1 pathway. To further validate the role of the cAMP/PKA/IRE1 pathway in hepatocyte ferroptosis, we treat HepG2 cells with PKA inhibitors (H-89), cAMP/PKA activators (dbcAMP), and IRE1 inhibitors (4μ8C), respectively. After inhibition of PKA and IRE1, the reduction of GPX4 (Figure 7A, B), NRF2 (Figure 7C), and FPN (Figure 7D), as well as the increase of TFR1 (Figure 7E) and DMT1 (Figure 7F) induced by Erastin, could be significantly restored. After activating cAMP/PKA, the recovery effect of Mel on GPX4, NRF2, FPN, TFR1, and DMT1 was significantly inhibited. These results suggested that Mel alleviated hepatocyte ferroptosis through the MT2/cAMP/PKA/IRE1 signaling pathway.

## Discussion

Apoptosis has been demonstrated to be the primary mode of hepatocyte death with the onset of NAFLD throughout the past few decades 24. Ferroptosis is a recently identified type of iron-dependent programmed cell death that is linked to the evolution of NAFLD, but the pathogenic relationship and mechanism between the two have not yet been fully uncovered 25. Finding effective interventions to resist hepatic ferroptosis and elucidating its mechanisms emerge as a potential avenue for treating NAFLD. Here, we report that Mel, a well-known antioxidant, inhibits hepatic ferroptosis in mice with NAFLD progression. Mechanistically, Mel can alleviate hepatic ferroptosis by inhibiting ER stress via the MT2/cAMP/PKA/IRE1 signaling pathway.

There are currently no approved pharmacological solutions for NAFLD as a global concern. Mel has previously been shown to prevent NAFLD and protect liver function in patients with diabetes and obesity 19, 26. In our study, we also found that Mel injection ameliorated HFD-induced global metabolic abnormalities, such as weight loss, lower TC levels, and restoration of dysregulated glucose homeostasis. Mel also significantly slowed the development of NAFLD, which included reducing liver steatosis, inflammation, and fibrosis. The mechanisms of Mel in improving NAFLD involve apoptosis signaling pathway 19, mitochondrial functions 27, oxidative stress pathways 28, autophagy 29, and inflammation 30. However, Mel may improve NAFLD in ways that go far beyond these. Apoptosis, necrosis, or pyrosis inhibition can only partially reduce liver damage in NASH 31, 32. Mel may treat NAFLD by participating in other forms of death in the liver.

Inhibition of hepatic ferroptosis is critical for NAFLD. For instance, ferroptosis suppression almost entirely protected hepatic necrotic death and decreased subsequent inflammatory response, whereas necroptosis inhibition did not stop the initiation of necrotic cell death 33. Ferroptosis is induced by intracellular iron accumulation and lipid peroxidation. Iron overload plays an important role in the onset of liver diseases 34. In this study, we found that Mel markedly restored Erastin- or PA-induced liver cell death. Furthermore, HFD significantly increased iron accumulation in the liver, and changes in proteins involved in iron transport and export in the liver were observed, including increases in TFR1 and DMT1, and decreases in FPN and Fth1. Supplementation with Mel reversed these effects.

Similar results were also observed *in vitro*. The ability of Mel to chelate iron, neutralize free radicals, and control the activity of the redox enzyme are three possible ways that it can prevent iron toxicity 35. The currently available evidence on how Mel affects iron transport is lacking. Our earlier research demonstrated that Mel can counteract the acute sleep deprivation-induced changes in transferrin expression in the hippocampus 15. Nonetheless, the mechanism of Mel is not fully understood in the current investigation, which is the limitation of the study. Lipid peroxidation is yet another crucial aspect of ferroptosis. Excess active iron donates electrons, generating reactive oxygen species (ROS) through the Fenton reaction 36, promoting lipid peroxidation and initiating ferroptosis. As a result of ferroptosis, lipid peroxidation gives rise to MDA, while GSH-Px and SOD are key antioxidant enzymes to remove excess MDA and ROS 37. Most importantly, the decline in GPX4 is the core regulator to neutralize lipid peroxidation products 38. We found that Mel treatment observably restored the levels of T-AOC, GSH-px, SOD, GPX4, KEAP1, NRF2, and HO-1. The process by which Mel counteracts hepatic lipid peroxidation is relatively well understood since Mel is a powerful free radical scavenger and antioxidant 15. Taken together, Mel inhibited hepatic ferroptosis by ameliorating iron homeostasis dysregulation and lipid peroxidation in the liver.

Next, we focused on the mechanism of Mel in ameliorating hepatic ferroptosis. Mel acts primarily through its membrane receptors, MT1 or MT2, in mammals. We found that Mel reversed HFD-decreased MT2 expression in the liver. The expression of MT1 remained unchanged. This is consistent with *in vitro* induction with PA or Erastin, and treatment with Mel or Fer-1. We further used Luzindole and 4P-PDOT to block the effect of Mel. Interestingly, the ameliorative effect of Mel on hepatic ferroptosis could be reversed by 4P-PDOT instead of Luzindole. Thus, MT2 mediates the effect of Mel on improving hepatic ferroptosis, including affecting iron ion transport and lipid peroxidation processes, at least in part. Evidence showing the role of Mel receptors in liver diseases is somewhat complicated and inadequate. As reported, Mel protected hepatic glycogen synthesis through MT2 in rats 39. However, MT1 but not MT2 knockout reduced bile duct injury and liver fibrosis in cholestatic liver injury 40. For one, MT2 is thought to play a more critical role in influencing metabolic processes such as glucose metabolism and insulin secretion 41. For another, the MT2 receptor prevents white blood cells from adhering and rolling, which may account for Mel's anti-inflammatory properties 42. Besides, depending on the content of their natural agonists or the length of exposure, MT1 and MT2 may experience varying desensitization or internalization, resulting in a diminished physiologic response 40, 43, 44. Exploration of the mechanisms underlying the distinct effects of MT1 and MT2 on liver phenotypes is both required and interesting. Moreover, Mel can directly pass through the cell membrane and bind to the nuclear receptors of the retinoid-related orphan nuclear hormone receptor family to exert physiological functions 45. Quinone reductase also acts as a binding site for Mel to balance the production of free radicals 46. It is not surprising that there may be Mel binding sites distinct from MT2 that mediate hepatic ferroptosis.

ER stress is considered a consequence of ferroptosis, whose activation may also contribute to ferroptosis. ER stress induces distinct cell death patterns through three major transmembrane receptors on the ER membrane [IRE1α, PERK, and activating transcription factor 6] 47. Next, we further explored the role of Mel in the relationship between hepatic ferroptosis and ER stress. Mel restored the hepatic expression levels of GRP78 Bip, p-PKA, p-IRE1, Xbp1, Xbp1s, and representative XBP1-dependent UPR target genes elevated by HFD. *In vitro*, Mel/MT2 significantly inhibited Erastin-activated p-PKA and p-IRE1 (Ser724). When ER occurs, IRE1 undergoes oligomerization and trans autophosphorylation upon separation from GRP78 Bip in the ER lumen, resulting in the activation of endoribonuclease and cleavage of its target gene, XBP1 mRNA, and subsequent unfolded UPR 48. Existing studies on ER stress and ferroptosis mainly focused on the PERK pathway 47. Here we show that Mel/MT2 signals affect the IRE1 pathway in the liver. Mel treatment inhibits IRE1, which is considered to improve acute pancreatitis 49, kidney injury 50, and osteoarthritis 51. Thus, Mel's inhibition of IRE1 may contribute to the amelioration of hepatic ferroptosis. MT2 is a G protein-coupled receptor, and its downstream signaling molecules, such as cAMP/PKA, are one of the most extensively studied receptor families 52. We found that p-PKA and p-IRE1 were co-expressed in the liver cytoplasm, which was enhanced by 4P-PDOT. Previous studies showed that activating IRE1α phosphorylation through PKA led to hepatic ER stress 53. Hence, we further investigated the role of PKA/IRE1-mediated ER stress in ferroptosis. Inhibition of PKA and IRE1 significantly ameliorated Erastin-induced hepatocyte ferroptosis, whereas the cAMP/PKA agonist blocked the improvement effect of Mel. Collectively, these suggest that the ER stress-mediated by the MT2/cAMP/PKA/IRE1 signaling pathway is critical for hepatic ferroptosis, which is also the main pathway for Mel to play an improving role.

## Conclusion

In summary, this study suggests iron homeostasis and lipid peroxidation in the liver jointly promote hepatic ferroptosis in HFD-induced NAFLD progression in mice. Exogenous Mel supplementation ameliorates hepatic ferroptosis through the MT2-cAMP-PKA-IRE1 axis-mediated ER stress. This implies that Mel serves as an effective therapeutic drug to inhibit hepatic ferroptosis in NAFLD.

## Supplementary Material

Supplementary materials and methods, table.

## Abbreviations

Acsl4: acyl-CoA synthetase long-chain family member 4
ALT: alanine aminotransferase
AST: aspartate aminotransferase
ATF4: activating transcription factor 4
BAT: brown adipose tissue
Bip: bound immunoglobulin
CAT: catalase
Chop: CCAAT/enhancer-binding protein homologous protein
Cox7b: cytochrome c oxidase subunit VIIb
DMT1: divalent metal transporter 1
Dnajb9: dnaJ heat shock protein family member B9
Edem1: er-degradation-enhancing α-mannosidase-like protein 1
Epi-WAT: epididymal white adipose tissue
ER: endoplasmic reticulum
FBS: fetal bovine serum
Fer-1: ferrostatin-1
FPN: ferroportin
Fth1: ferritin heavy chain 1
GSH-Px: glutathione peroxidase
HFD: high-fat diet
IHC: immunohistochemistry
IRE1: inositol-requiring enzyme 1
Mel: melatonin
MT1: melatonin receptor type 1A
MT2: melatonin receptor type 1B
MDA: malondialdehyde
NAFLD: non-alcoholic fatty liver disease
NASH: nonalcoholic steatohepatitis
NCD: normal chow diet
PA: palmitic acid
PERK: protein kinase R-like endoplasmic reticulum kinase
Pgc-1α: peroxisome proliferator-activated receptor-γ coactivator-1α
PKA: protein kinase A
ROS: reactive oxygen species
Sec61a1: SEC61 transposon α1 subunit
SEM: standard error of mean
SOD: superoxide dismutase
T-AOC: total antioxidant capability
TC: total cholesterol
TFR1: transferrin receptor 1
TG: triglyceride
URP: unfolded protein response
Vis-WAT: visceral white adipose tissue
XBP1: X-box binding protein 1

## References

[1] Younossi Z, Anstee QM, Marietti M, Hardy T, Henry L, Eslam M (2018). Global burden of NAFLD and NASH: trends, predictions, risk factors and prevention. Nat Rev Gastroenterol Hepatol.

[2] Yip TC, Lee HW, Chan WK, Wong GL, Wong VW (2022). Asian perspective on NAFLD-associated HCC. J Hepatol.

[3] Ji J, Wu L, Wei J, Wu J, Guo C (2023). The gut microbiome and ferroptosis in MAFLD. J Clin Transl Hepatol.

[4] Zhou J, Zhou F, Wang W, Zhang XJ, Ji YX, Zhang P (2020). Epidemiological features of NAFLD from 1999 to 2018 in China. Hepatology.

[5] Tang D, Chen X, Kang R, Kroemer G (2021). Ferroptosis: molecular mechanisms and health implications. Cell Res.

[6] Chen X, Comish PB, Tang D, Kang R (2021). Characteristics and biomarkers of ferroptosis. Front Cell Dev Biol.

[7] Zheng J, Conrad M (2020). The metabolic underpinnings of ferroptosis. Cell Metab.

[8] Wu S, Yang J, Sun G, Hu J, Zhang Q, Cai J (2021). Macrophage extracellular traps aggravate iron overload-related liver ischaemia/reperfusion injury. Br J Pharmacol.

[9] Qi J, Kim JW, Zhou Z, Lim CW, Kim B (2020). Ferroptosis affects the progression of nonalcoholic steatohepatitis via the modulation of lipid peroxidation-mediated cell death in mice. Am J Pathol.

[10] Zhang X, Li W, Ma Y, Zhao X, He L, Sun P (2021). High-fat diet aggravates colitis-associated carcinogenesis by evading ferroptosis in the ER stress-mediated pathway. Free Radic Biol Med.

[11] Zhang X, Jiang L, Chen H, Wei S, Yao K, Sun X (2022). Resveratrol protected acrolein-induced ferroptosis and insulin secretion dysfunction via ER-stress- related PERK pathway in MIN6 cells. Toxicology.

[12] Jiang D, Niwa M, Koong AC (2015). Targeting the IRE1α-XBP1 branch of the unfolded protein response in human diseases. Semin Cancer Biol.

[13] Wei R, Zhao Y, Wang J, Yang X, Li S, Wang Y (2021). Tagitinin C induces ferroptosis through PERK-Nrf2-HO-1 signaling pathway in colorectal cancer cells. Int J Biol Sci.

[14] Liu J, Clough SJ, Hutchinson AJ, Adamah-Biassi EB, Popovska-Gorevski M, Dubocovich ML (2016). MT1 and MT2 nelatonin receptors: A therapeutic perspective. Annu Rev Pharmacol Toxicol.

[15] Wang X, Wang Z, Cao J, Dong Y, Chen Y (2021). Melatonin alleviates acute sleep deprivation-induced memory loss in mice by suppressing hippocampal ferroptosis. Front Pharmacol.

[16] Guohua F, Tieyuan Z, Xinping M, Juan X (2021). Melatonin protects against PM2.5-induced lung injury by inhibiting ferroptosis of lung epithelial cells in a Nrf2-dependent manner. Ecotoxicol Environ Saf.

[17] Gao T, Wang Z, Dong Y, Cao J, Lin R, Wang X (2019). Role of melatonin in sleep deprivation-induced intestinal barrier dysfunction in mice. J Pineal Res.

[18] Xu P, Wang J, Hong F, Wang S, Jin X, Xue T (2017). Melatonin prevents obesity through modulation of gut microbiota in mice. J Pineal Res.

[19] Li DJ, Tong J, Li YH, Meng HB, Ji QX, Zhang GY (2019). Melatonin safeguards against fatty liver by antagonizing TRAFs-mediated ASK1 deubiquitination and stabilization in a β-arrestin-1 dependent manner. J Pineal Res.

[20] Yin J, Li Y, Han H, Chen S, Gao J, Liu G (2018). Melatonin reprogramming of gut microbiota improves lipid dysmetabolism in high-fat diet-fed mice. J Pineal Res.

[21] Zhang YH, Wang DW, Xu SF, Zhang S, Fan YG, Yang YY (2018). α-Lipoic acid improves abnormal behavior by mitigation of oxidative stress, inflammation, ferroptosis, and tauopathy in P301S Tau transgenic mice. Redox Biol.

[22] Kleiner DE, Brunt EM, Van Natta M, Behling C, Contos MJ, Cummings OW (2005). Design and validation of a histological scoring system for nonalcoholic fatty liver disease. Hepatology.

[23] Mao T, Shao M, Qiu Y, Huang J, Zhang Y, Song B (2011). PKA phosphorylation couples hepatic inositol-requiring enzyme 1alpha to glucagon signaling in glucose metabolism. Proc Natl Acad Sci U S A.

[24] Tanaka S, Hikita H, Tatsumi T, Sakamori R, Nozaki Y, Sakane S (2016). Rubicon inhibits autophagy and accelerates hepatocyte apoptosis and lipid accumulation in nonalcoholic fatty liver disease in mice. Hepatology.

[25] Feng G, Byrne CD, Targher G, Wang F, Zheng MH (2022). Ferroptosis and metabolic dysfunction-associated fatty liver disease: Is there a link?. Liver Int.

[26] Sun H, Wang X, Chen J, Song K, Gusdon AM, Li L (2016). Melatonin improves non-alcoholic fatty liver disease via MAPK-JNK/P38 signaling in high-fat-diet-induced obese mice. Lipids Health Dis.

[27] Zhou H, Du W, Li Y, Shi C, Hu N, Ma S (2018). Effects of melatonin on fatty liver disease: The role of NR4A1/DNA-PKcs/p53 pathway, mitochondrial fission, and mitophagy. J Pineal Res.

[28] Joshi A, Upadhyay KK, Vohra A, Shirsath K, Devkar R (2021). Melatonin induces Nrf2-HO-1 reprogramming and corrections in hepatic core clock oscillations in Non-alcoholic fatty liver disease. FASEB J.

[29] Stacchiotti A, Grossi I, García-Gómez R, Patel GA, Salvi A, Lavazza A (2019). Melatonin effects on non-alcoholic fatty liver disease are related to MicroRNA-34a-5p/Sirt1 axis and autophagy. Cells.

[30] Saha M, Manna K, Das Saha K (2022). Melatonin suppresses NLRP3 inflammasome activation via TLR4/NF-κB and P2X7R signaling in high-fat diet-induced murine NASH model. J Inflamm Res.

[31] Xu B, Jiang M, Chu Y, Wang W, Chen D, Li X (2018). Gasdermin D plays a key role as a pyroptosis executor of non-alcoholic steatohepatitis in humans and mice. J Hepatol.

[32] Anstee QM, Concas D, Kudo H, Levene A, Pollard J, Charlton P (2010). Impact of pan-caspase inhibition in animal models of established steatosis and non-alcoholic steatohepatitis. J Hepatol.

[33] Tsurusaki S, Tsuchiya Y, Koumura T, Nakasone M, Sakamoto T, Matsuoka M (2019). Hepatic ferroptosis plays an important role as the trigger for initiating inflammation in nonalcoholic steatohepatitis. Cell Death Dis.

[34] Pan Q, Luo Y, Xia Q, He K (2021). Ferroptosis and liver fibrosis. Int J Med Sci.

[35] Yang J, Tang Q, Zeng Y (2022). Melatonin: Potential avenue for treating iron overload disorders. Ageing Res Rev.

[36] Xie LH, Fefelova N, Pamarthi SH, Gwathmey JK (2022). Molecular mechanisms of ferroptosis and relevance to cardiovascular disease. Cells.

[37] Dixon SJ, Lemberg KM, Lamprecht MR, Skouta R, Zaitsev EM, Gleason CE (2012). Ferroptosis: an iron-dependent form of nonapoptotic cell death. Cell.

[38] Guo M, Zhu Y, Shi Y, Meng X, Dong X, Zhang H (2022). Inhibition of ferroptosis promotes retina ganglion cell survival in experimental optic neuropathies. Redox Biol.

[39] Li T, Ni L, Zhao Z, Liu X, Lai Z, Di X (2018). Melatonin attenuates smoking-induced hyperglycemia via preserving insulin secretion and hepatic glycogen synthesis in rats. J Pineal Res.

[40] Wu N, Carpino G, Ceci L, Baiocchi L, Francis H, Kennedy L (2022). Melatonin receptor 1A, but not 1B, knockout decreases biliary damage and liver fibrosis during cholestatic liver injury. Hepatology.

[41] Karamitri A, Jockers R (2019). Melatonin in type 2 diabetes mellitus and obesity. Nat Rev Endocrinol.

[42] Dubocovich ML, Markowska M (2005). Functional MT1 and MT2 melatonin receptors in mammals. Endocrine.

[43] Gerdin MJ, Masana MI, Dubocovich ML (2004). Melatonin-mediated regulation of human MT(1) melatonin receptors expressed in mammalian cells. Biochem Pharmacol.

[44] Gerdin MJ, Masana MI, Rivera-Bermúdez MA, Hudson RL, Earnest DJ, Gillette MU (2004). Melatonin desensitizes endogenous MT2 melatonin receptors in the rat suprachiasmatic nucleus: Relevance for defining the periods of sensitivity of the mammalian circadian clock to melatonin. Faseb j.

[45] Zhao CN, Wang P, Mao YM, Dan YL, Wu Q, Li XM (2019). Potential role of melatonin in autoimmune diseases. Cytokine Growth Factor Rev.

[46] Boutin JA (2016). Quinone reductase 2 as a promising target of melatonin therapeutic actions. Expert Opin Ther Targets.

[47] Zhang J, Guo J, Yang N, Huang Y, Hu T, Rao C (2022). Endoplasmic reticulum stress-mediated cell death in liver injury. Cell Death Dis.

[48] Chen Y, Brandizzi F (2013). IRE1: ER stress sensor and cell fate executor. Trends Cell Biol.

[49] Zhao Q, Tang X, Huang J, Li J, Chen Q, Sun Y (2018). Melatonin attenuates endoplasmic reticulum stress in acute pancreatitis. Pancreas.

[50] Aouichat S, Navarro-Alarcon M, Alarcón-Guijo P, Salagre D, Ncir M, Zourgui L (2021). Melatonin improves endoplasmic reticulum stress-mediated IRE1α pathway in Zücker diabetic fatty rat. Pharmaceuticals (Basel).

[51] Qin K, Tang H, Ren Y, Yang D, Li Y, Huang W (2022). Melatonin promotes sirtuin 1 expression and inhibits IRE1α-XBP1S-CHOP to reduce endoplasmic reticulum stress-mediated apoptosis in chondrocytes. Front Pharmacol.

[52] Weis WI, Kobilka BK (2018). The molecular basis of G protein-coupled receptor activation. Annu Rev Biochem.

[53] Liu X, Wang K, Wang L, Kong L, Hou S, Wan Y (2022). Hepatocyte leukotriene B4 receptor 1 promotes NAFLD development in obesity. Hepatology.

