# Supplementary material for: Melatonin Ameliorates Hepatic Ferroptosis in NAFLD by Inhibiting ER Stress via the MT2/cAMP/PKA/IRE1 Signaling Pathway
Source: Int J Biol Sci. 2023 Jul 31;19(12):3937–50. doi: 10.7150/ijbs.85883 (PMC10411470; doi:10.7150/ijbs.85883)
Supplement: Supplementary file 1 — Supplementary materials and methods, table. [file ijbsv19p3937s1.pdf]

Supplementary Material

Materials and Methods

2.6. Immunohistochemistry and immunofluorescence

Paraffin-embedded liver slices were treated with primary antibodies overnight at 4°C for immunohistochemistry staining, including mouse EGF-like module-containing mucin-like hormone receptor-like 1 (F4/80, 1:200; Proteintech Group, Inc, Rosemont, USA), Mel receptor type 1A (MT1, 1:200; Abcam, Cambridge, UK), and Mel receptor type 1B (MT2, 1:200; Abcam, Cambridge, UK). The sections were then treated with biotin-conjugated goat anti-rabbit IgG (1:300; Santa Cruz) at room temperature for 2 hours after being rinsed with 0.01 M PBS (pH, 7.4). Following a wash, they were incubated for 1.5 hours at room temperature with streptavidin-horseradish peroxidase (1:300; Vector Laboratories, CA, USA). Sections were treated with hematoxylin and fixed after being incubated for 10 minutes in 0.01 M PBS containing 0.05% diaminobenzidine (DAB, Sigma, St. Louis, MO, USA) and 0.003% H_2O_2. In every instance, control formulations devoid of primary antibodies were evaluated. The immunoreactive area's cytoplasm or nucleus was yellow-brown in color.

For the immunofluorescence assay, liver slices and HepG2 cells were treated overnight at 4°C with transferrin receptor 1 (TFR1, 1:100; Proteintech Group, Inc, Rosemont, USA) and glutathione peroxidase 4 (GPX4, 1:100; Proteintech Group, Inc, Rosemont, USA). Besides, HepG2 cells were treated overnight at 4°C with p-IRE1 (phospho S724, 1:100; Bioss Antibody, Beijing, China). The sections were then

washed in 0.01 M PBST (pH 7.4) and treated for 2 hours at room temperature with biotinylated goat anti-rabbit or mouse IgG (1:300; Beyotime, Shanghai, China). After washing, tissues or cells were treated in AF-594-streptavidin or AF-488-streptavidin (1:100; Sigma, St. Louis, MO, USA) for 1.5 hours at room temperature. Following that, the sections were washed with 0.01 M PBST (pH 7.4). Finally, DAPI (1 μg/mL, C0065, Solarbio, Beijing, China) is used to stain the nuclei for 15 minutes. A fluorescence microscope (BX51, Olympus, Tokyo, Japan) was used to observe the location and distribution of immunoreactive substances in liver tissue or cells.

2.7. Quantitative real-time (RT)-PCR analysis

The first-strand cDNA synthesis kit (R312-01; Vazyme, Nanjing, China) was used to perform reverse transcription on the total mRNA in order to produce cDNA. The AceQ qPCR SYBR green master mix (Q111-02; Vazyme) was then used to perform RT-PCR amplification with chosen gene primers. The primers were listed in Table S1. The expression levels of Gapdh were used to normalize relative mRNA levels.

2.8. Western blot assay

8-12% sodium dodecyl sulfate-polyacrylamide gel electrophoresis (SDS-PAGE) was used to separate the protein samples, and the results were electro-imprinted onto polyvinylidene fluoride (PVDF) membranes (Millipore, Billerica, MA, USA). PVDF membranes were then sealed for 1.5 hours with TBST containing 5% skim milk. After that, they were incubated overnight at 4°C with primary antibodies: p-P65 (1:1000, CST), NOD-like receptor thermal protein domain associated protein 3 (NLRP3, 1:1000; CST), α-smooth muscle actin (α-SMA, 1:1000; 14395-1-AP, Proteintech),

TFR1 (1:2000; 66180-1-Ig, Proteintech), Ferroportin (FPN, 1:1000; NBP1-21502SS, Novus), divalent metal transporter 1 (DMT1, 1:1000; NBP1-91840, Novus), GPX4 (1:1000; 67763-1-Ig, Proteintech), inducible nitric oxide synthase (iNOS, 1:1000; CST), Kelch-1ike ECH- associated protein l (KEAP1, 1:1000; Abmart), Nuclear Factor erythroid 2-related factor 2 (NRF2, 1:1000; CST), MT1 (1:1000, ab203038, Abcam), MT2 (1:1000, ab203346, Abcam), p-PKA (1:5000, ab75991, Abcam), p-IRE1 (1:1000, bs-16698R, Bioss Antibodies), PERK (1:1000, TA5304, Abmart) and β-actin (1:8000; Proteintech). The membranes were rinsed with TBST and treated for 1.5 hours with horseradish peroxidase-conjugated goat anti-rabbit IgG (1:8000; CW0103, COWIN) or goat anti-mouse IgG (1:8000; CW0102, COWIN). The embossed bands were scanned and examined, and the IOD was calculated using Image J (Scion Corp., Frederick, MD, USA). The relative protein level in the Ctrl group in vivo or in the control cells in vitro was defined as 100%. The protein level was adjusted to the density ratio of β-actin.

2.9. Cell culture and treatment

The effect of palmitic acid (PA) or Erastin on the viability of HepG2 cells was examined using the 3-(4,5-dimethylthiazol-2-yl)-2,5-diphenyltetrazolium bromide (MTT) assay. In a 96-well plate, cells were seeded and incubated for 6 hours at 37°C with 5% CO_2. After that, we switched to basal medium and proceeded to cultivate the cells for another 12 hours. Following a 30-minute pretreatment with PA (HY-N0830; MCE, China) or Erastin (E7781; Sigma, USA), the cells were subjected to varying dosages of Fer-1 (SML0583; Sigma, USA) or Mel (M5250; Sigma, USA) for 24

hours. MTT solution (5 mg/mL in PBS, Sigma, St. Louis, MO, USA) was added afterward, and the mixture was then incubated for 4 hours. The crystals were dissolved in 150 μL of dimethyl sulfoxide (DMSO; Sigma, St. Louis, MO, USA) after the supernatant was removed. The microplate reader was used to find the absorbance at 490 nm (Synergy HT. BioTek, Winooski, VT, USA).

Ferroptosis inducer Erastin (E7781; Sigma, USA), Fer-1 (SML0583; Sigma, USA), MT1-selective Mel receptor antagonist Luzindole (HY-101254; MCE, China), MT2-selective Mel receptor antagonist 4P-PDOT (HY-100609; MCE, China), PKA inhibitor H-89 (HY-15979A; MCE, China), IRE1 inhibitor 4μ8C (HY-19707; MCE, China), c-AMP and PKA agonist Dibutyryl-cAMP (db-cAMP, HY-B0764; MCE, China) separately dissolved in DMSO. Dissolve Mel (M5250; Sigma, USA) in anhydrous ethanol. Cells were pretreated with Luzindole (1 μM), 4P-PDOT (1 μM), H-89 (1 μM), 4μ8C (1μM), or db-cAMP (1 μM) for 30 min, followed by pretreated with Mel (1 μM) or Fer-1 (2 μM) for 30 min prior to 24 hours exposure to PA (250 μM) or Erastin (1 μM). On a 6-well plate, the protein content was evaluated.

Cells were plated onto slide glass (801010, Nest, Jiangsu, China), grown, and treated as previously mentioned. 95% ethanol was used to immobilize the cells. After washing with PBS three times, 5% goat serum was used to seal at 37°C for 30 minutes. Then, cells were incubated with p-IRE1 (1:100) or GPX4 (1:100) overnight at 4°C. The precise procedure is identical to the immunofluorescence assay for the slices of liver tissue described above.

Cells were treated overnight at 4°C in murine anti-p-PKA (1:100) firstly for

double labeling immunofluorescence. Wash the cells, then incubate them for 2 hours at room temperature in biotinylated goat anti-mouse IgG (1:300; Beyotime, Shanghai, China). Following a wash, cells were incubated for 1.5 hours at room temperature with AF-594-streptavidin (1:100; Sigma, Louis, MO, USA). Prior to being incubated with rabbit anti-p-IRE 1 (1:100) for the entire overnight period at 4°C, cells were washed and blocked with 5% goat serum for 30 minutes at 37°C. Wash the cells, then incubate them for 2 hours at room temperature in biotinylated goat anti-rabbit IgG (1:300; Beyotime, Shanghai, China). Following a wash, cells were incubated for 1.5 hours at room temperature with AF-488-streptavidin (1:100; Sigma, Louis, MO, USA). Finally, the nuclei are stained for 15 minutes with DAPI (1μg/mL, C0065, Solarbio, Beijing, China). A fluorescent microscope was used to examine the localization and distribution of immunoreactive positive materials in HepG2 cells (BX51, Olympus, Tokyo, Japan).

Table S1. Sequences of primers used for RT-PCR.

**Gene**	**Primer sequence (5′ to 3′)**		**Product size**	**Accession**
α-SMA	F: GTCCCAGACATCAGGGAGTAA	R: TCGGATACTTCAGCGTCAGGA	102	NM_007392.3
Tgf-β1	F: CTCCCGTGGCTTCTAGTGC	R: GCCTTAGTTTGGACAGGATCTG	133	NM_011577.2
Collagen-1	F: CATGTTCAGCTTTGTGGACCT	R: GCAGCTGACTTCAGGGATGT	94	NM_007742.4
Ccl2	F: TACAAGAGGATCACCAGCAGC	R: ACCTTAGGGCAGATGCAGTT	181	NM_011333.3
Ccl5	F: TGCTGCTTTGCCTACCTCTC	R: TCTTCTCTGGGTTGGCACAC	139	NM_013653.3
Ilb1	F: CCGTGGACCTTCCAGGATGA	R: GGGAACGTCACACACCAGCA	117	NM_008361.4
Fpn1	F: TGGAACTCTATGGAAACAGCCT	R: TGGCATTCTTATCCACCCAGT	111	NM_016917.2
Dmt1	F: TACCTAGACCCAGGAAACATCG	R: CACTCCAAGTCTCGCTGCAA	132	NM_008732.2
Ftl1	F: CGTCAGAATTATTCCACCGAGG	R: GCCACGTCATCCCGATCAAA	116	NM_010240.2
Fth1	F: CAAGTGCGCCAGAACTACCA	R: ACAGATAGACGTAGGAGGCATAC	88	NM_010239.2
Alox5ap	F: GCATGAAAGCAAGGCGCATAA	R: GGTACGCATCTACGCAGTTCT	101	NM_009663.2
Acsl4	F: CCTGAGGGGCTTGAAATTCAC	R: GTTGGTCTACTTGGAGGAACG	88	NM_019477.3
Atf4	F: CTCTTGACCACGTTGGATGAC	R: CAACTTCACTGCCTAGCTCTAAA	226	NM_009716.3
Pgc-1α	F: TATGGAGTGACATAGAGTGTGCT	R: CCACTTCAATCCACCCAGAAAG	134	NM_008904.3
Ogdh	F: GTTTCTTCAAACGTGGGGTTCT	R: GCATGATTCCAGGGGTCTCAAA	232	NM_001361905.1
Idh3b	F: TGGAGAGGTCTCGGAACATCT	R: AGCCTTGAACACTTCCTTGAC	150	NM_130884.4
Uqcrc1	F: AGACCCAGGTCAGCATCTTG	R: GCCGATTCTTTGTTCCCTTGA	174	NM_025407.2
Cox7b	F: TTGCCCTTAGCCAAAAACGC	R: TCATGGAAACTAGGTGCCCTC	101	NM_025379.2
Xbp1	F: AAGAACACGCTTGGGAATGG	R: ACTCCCCTTGGCCTCCAC	67	NM_013842.3
Xbp1s	F: GAGTCCGCAGCAGGTG	R: GTGTCAGAGTCCATGGGA	65	NM_001271730.1
Dnajb9	F: CTCCACAGTCAGTTTTCGTCTT	R: GGCCTTTTTGATTTGTCGCTC	128	NM_013760.4
Edem1	F: GGGGCATGTTCGTCTTCGG	R: CGGCAGTAGATGGGGTTGAG	79	NM_138677.2
Sec61a1	F: GGAAGTCATCAAGCCATTCTGT	R: GCATCCAGTAGAACGGGTCAG	179	NM_016906.4
Bip	F: ACTTGGGGACCACCTATTCCT	R: ATCGCCAATCAGACGCTCC	134	NM_022310.3
Chop	F: CTGGAAGCCTGGTATGAGGAT	R: CAGGGTCAAGAGTAGTGAAGGT	121	NM_007837.4
Gapdh	F: CCGAGAATGGGAAGCTTGTC	R: TTCTCGTGGTTCACACCCATC	232	NM_008084.4

F: Forward primer; R: Reverse primer
